# Supplementary material for: GLUL Ablation Can Confer Drug Resistance to Cancer Cells via a Malate-Aspartate Shuttle-Mediated Mechanism
Source: Cancers (Basel). 2019 Dec 5;11(12):1945. doi: 10.3390/cancers11121945 (PMC6966511; doi:10.3390/cancers11121945)
Supplement: Supplementary file 1 [file cancers-11-01945-s001.pdf]

cancers

GLUL Ablation Can Confer Drug Resistance to Cancer Cells via a Malate-Aspartate Shuttle-Mediated Mechanism

Magesh Muthu, Ranjeet Kumar, Azharuddin Sajid Syed Khaja, Jonathan D. Gilthorpe, Jenny L. Persson and Anders Nordström

Supplementary information:

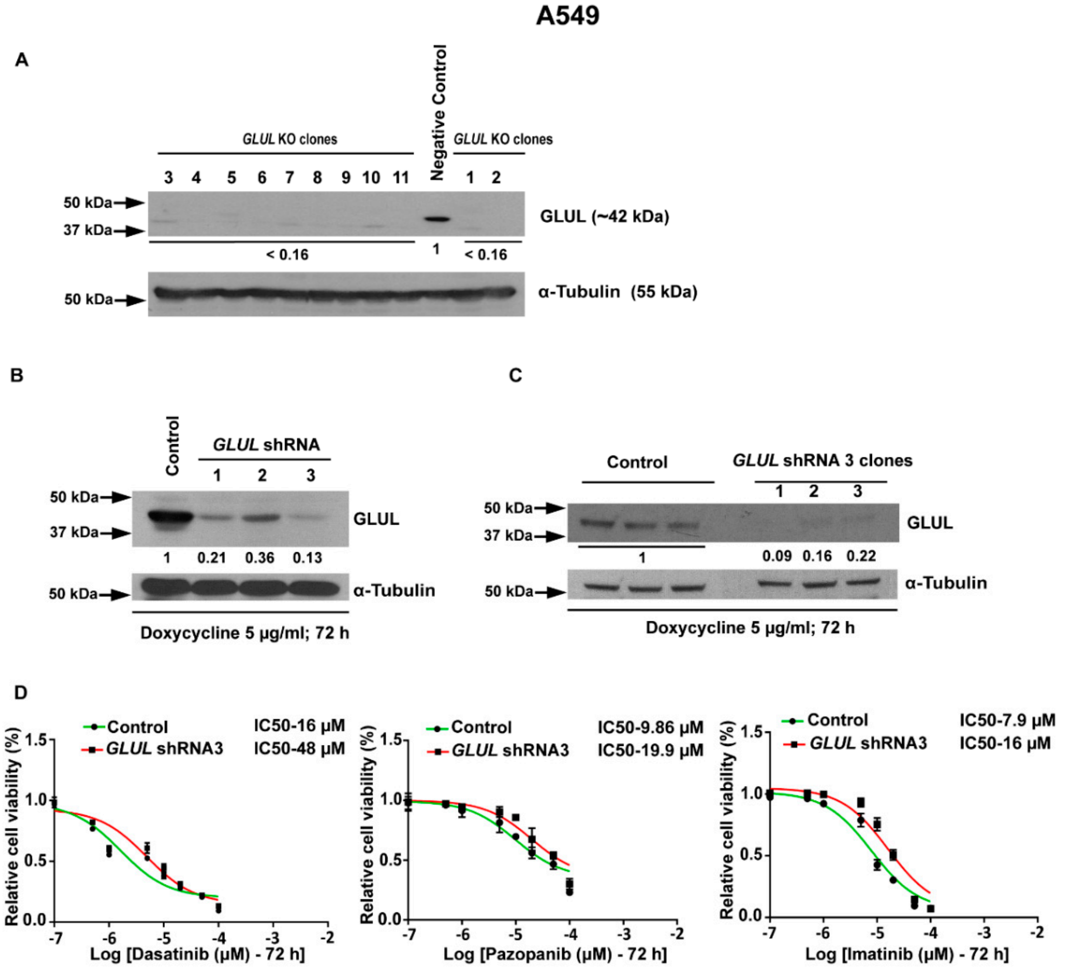

Figure 1. *GLUL* KO/KD induces drug resistance. (**A**) Western blot shows screening of CRISPR/Cas9 mediated *GLUL* KO clones. (**B**,**C**) A549 cells either transfected with non-target control or with doxycycline inducible shRNA (1, 2, 3) targeting *GLUL*. Subsequently cell lysates were analysed by western blot (WB) as in methods for GLUL expression levels and individual clones were screened and selected as shown above. The western blot membranes were subsequently probed with anti-tubulin antibody to assess equal loading. The presence of GLUL protein is indicated in the right side of each blot. The signals for the GLUL and α-tubulin proteins were quantified by densitometry, and the numbers below GLUL blot indicate levels of GLUL protein in each lane following normalization of the signals with actin levels. For the sake of comparison, the signal intensity for GLUL in control lane was assigned an arbitrary value of 1. Approximate location of various molecular weight is indicated on left side of each blot. kDa, kilodalton. (**D**) Upon doxycycline induction, A549 *GLUL* shRNA3 cl3

cells were treated with respective drugs of various concentrations as indicated and cell viability were assessed by MTS assay after 72 h. Further, IC_{50} were determined as indicated. The standard error (SE) bars in cell viability curve represent means of three independent experiments.

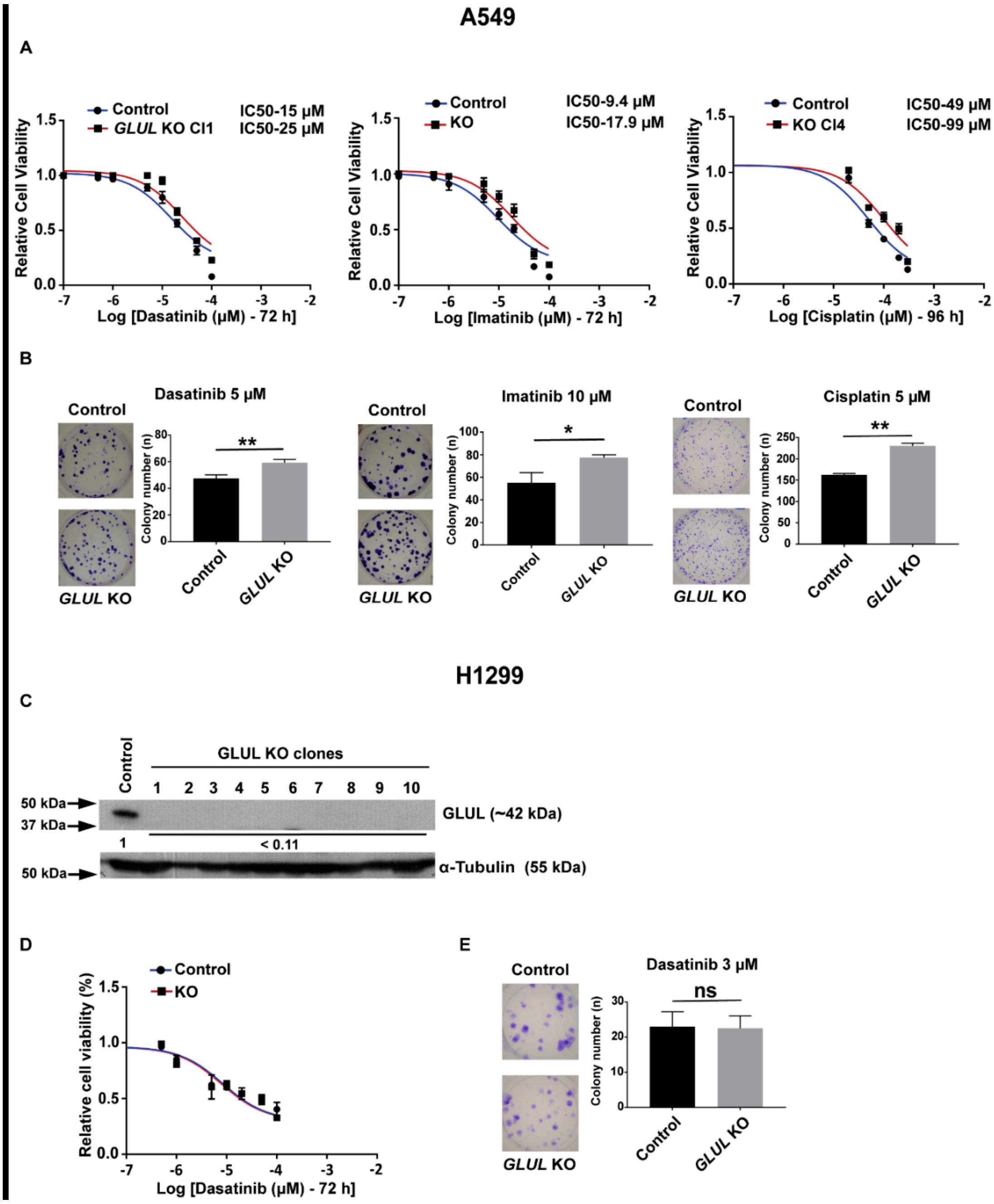

Figure 2. KO cells display drug resistance in A549 but not in H1299 cells. (**A**,**D**) A549 & H1299 cells were either transfected with negative control or with LentiviralCRISPR/Cas9 coupled *GLUL* guide RNA. Subsequently cells were treated with indicated chemotherapeutic drugs and cell viability was analysed by MTS assay after 72–96 h. IC_{50} for each drug was determined as indicated. (**B**,**E**) A549 & H1299 control and *GLUL* KO cells were treated with the indicated drugs and subjected to clonogenic assay. Histograms represent total number of colonies counted and representative images of crystal violet stained cells are shown. The standard error (SE) bars in cell viability assay and clonogenic assay represent means of three independent experiments. (**C**) H1299 cells were either transfected with negative control or with LentiviralCRISPR/Cas9 coupled *GLUL* guide RNA. Subsequently cell lysates were analysed by western blot (WB) as in methods for GLUL expression levels and individual clones were screened and selected as shown above. The western blot membranes were subsequently probed with anti-tubulin antibody to assess equal loading. The presence of GLUL and tubulin protein is

indicated in the right side of each blot. The presence of GLUL protein is indicated in the right side of each blot. The signals for the GLUL and α-tubulin proteins were quantified by densitometry, and the numbers below GLUL blot indicate levels of GLUL protein in each lane following normalization of the signals with actin levels. For the sake of comparison, the signal intensity for GLUL in control lane was assigned an arbitrary value of 1. Approximate location of various molecular weight is indicated on left side of each blot. kDa, kilodalton. The data shown as mean ± SEM; p-values were determined using a two-tailed unpaired t-test; ** $p \leq 0.004$, * $p \leq 0.01$, ns – not significant.

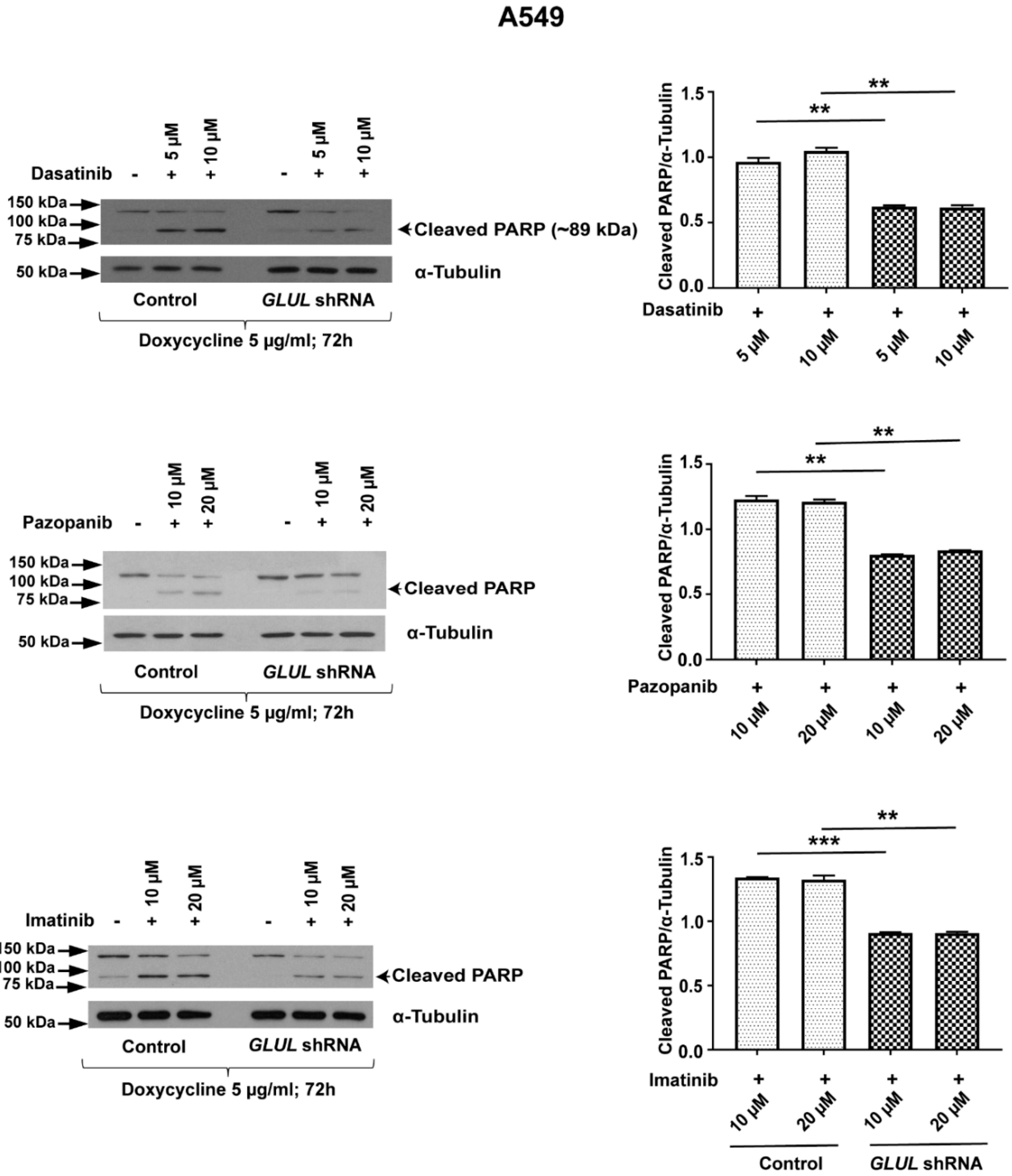

Figure 3. A549 *GLUL* KD exhibit less or no PARP cleavage. A549 Control (Non-target) or *GLUL* KD (*GLUL* shRNA3-cl3) cells were treated with doxycycline at a concentration of 5 µg/mL for 72 h and then were either treated with DMSO (control) or treated with chemotherapeutic drugs as mentioned for 12 h. Protein levels of cleaved PARP were assessed by western blot analysis. Subsequently, the membranes were probed with anti-tubulin antibody to assess equal loading. The presence of GLUL and tubulin protein is indicated in the right side of each blot. Approximate location of various molecular weight is indicated on left side of each blot. Quantification of protein levels is presented on ride side of each western blot. kDa, kilodalton.

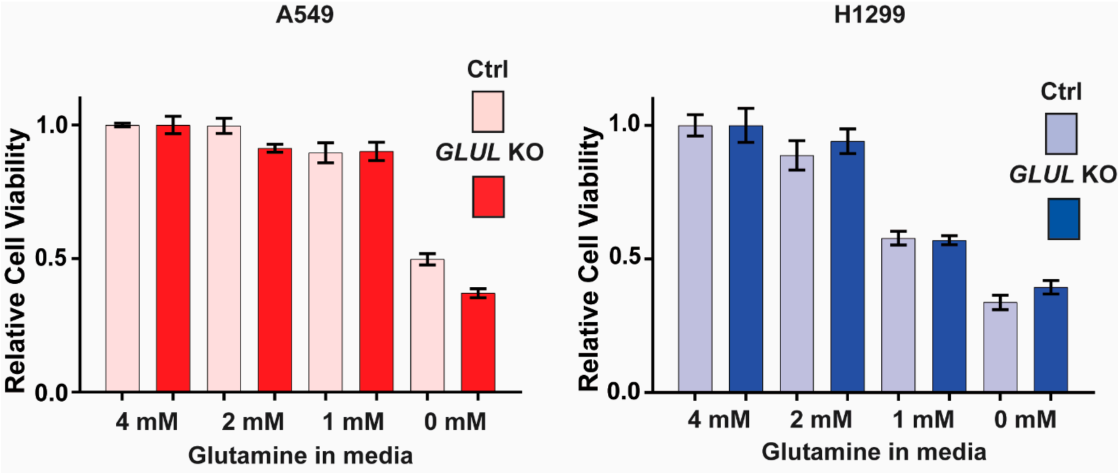

Figure 4. Reduced glutamine do not correlates with drug resistance in *GLUL* KO cells. A549 & H1299 (Control and *GLUL* KO) cells were cultured in various concentraions glutamine as indicated for 72 h and cell viabilty was analysed by MTS assay. Data for each pair of cell lines (Control/GLUL KO) was normalized to the data for the highest concetration of gluatmine (4 mM) as shown above. Data shown as ± standard deviation. The standard deviation (SD) bars in cell viability curve represent means of three independent experiments.

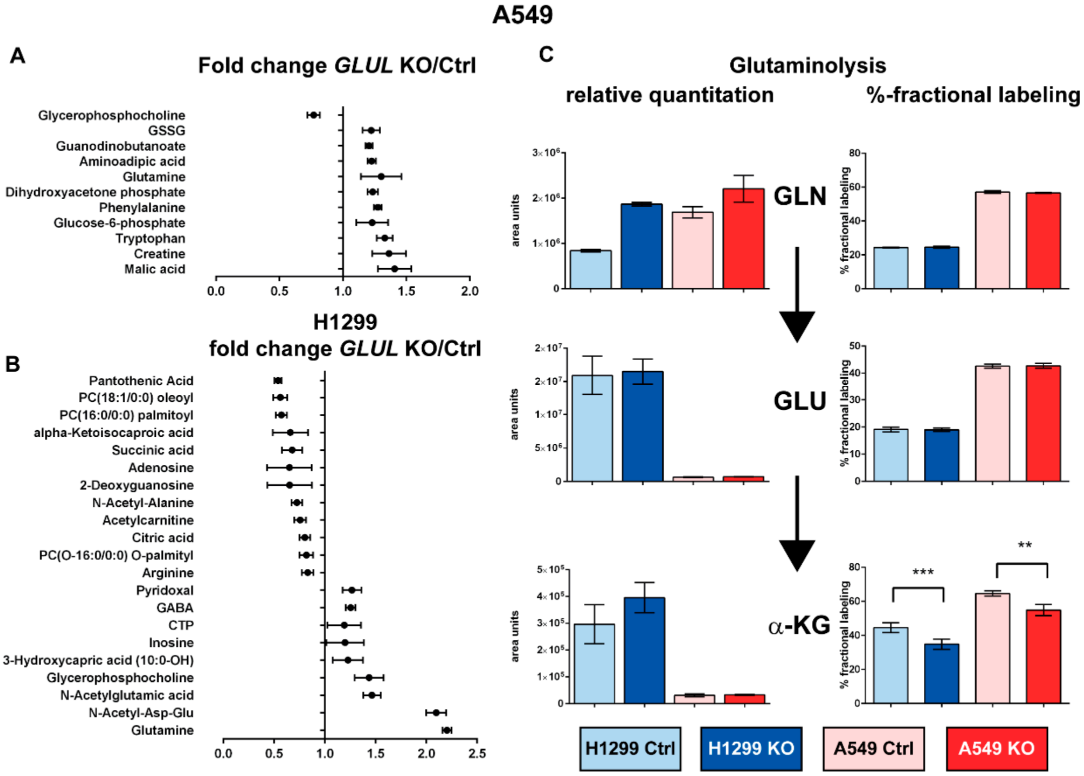

Figure 5. Targeted metabolomics reveals glutamine accumulation in resistant cells without increased glutamine uptake or flux through the glutaminolytic pathway. (**A**) Fold change for metabolites that displayed significant difference between *GLUL* KO/Control A549 drug resistant cells. (**B**) Fold change for metabolites that displayed significant difference between *GLUL* KO/Control H1299 drug sensitive cells. (**C**) Left, relative quantitation (area units) for glutamine (GLN), glutamate (GLU) and α-ketoglutarate (α-KG). Right, %-fractional labeling obtained through feeding $^{13}C_5$-glutamine to the cells. Data shown as ± standard deviation; p-values were determined using a two-tailed unpaired t-test; *** $p \leq 0.001$; ** $p \leq 0.01$.

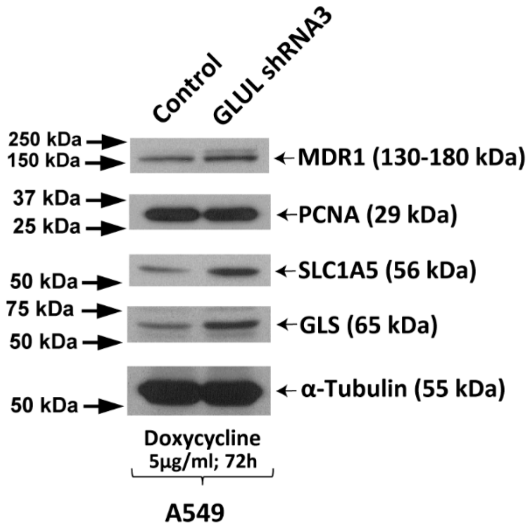

Figure 6. Reduced *GLUL* expression induces glutaminolysis. A549 Control (Non-target) or *GLUL* KD (*GLUL* shRNA3 cl3) cells were treated with doxycycline at a concentration of 5 µg/mL for 72 h and various Protein expressions were assessed by western blot analysis as shown above. Subsequently, the membranes were probed with anti-tubulin antibody to assess equal loading. The presence of various proteins is indicated in the right side of each blot and approximate location of various molecular weight is indicated on left side of each blot. kDa, kilodalton.

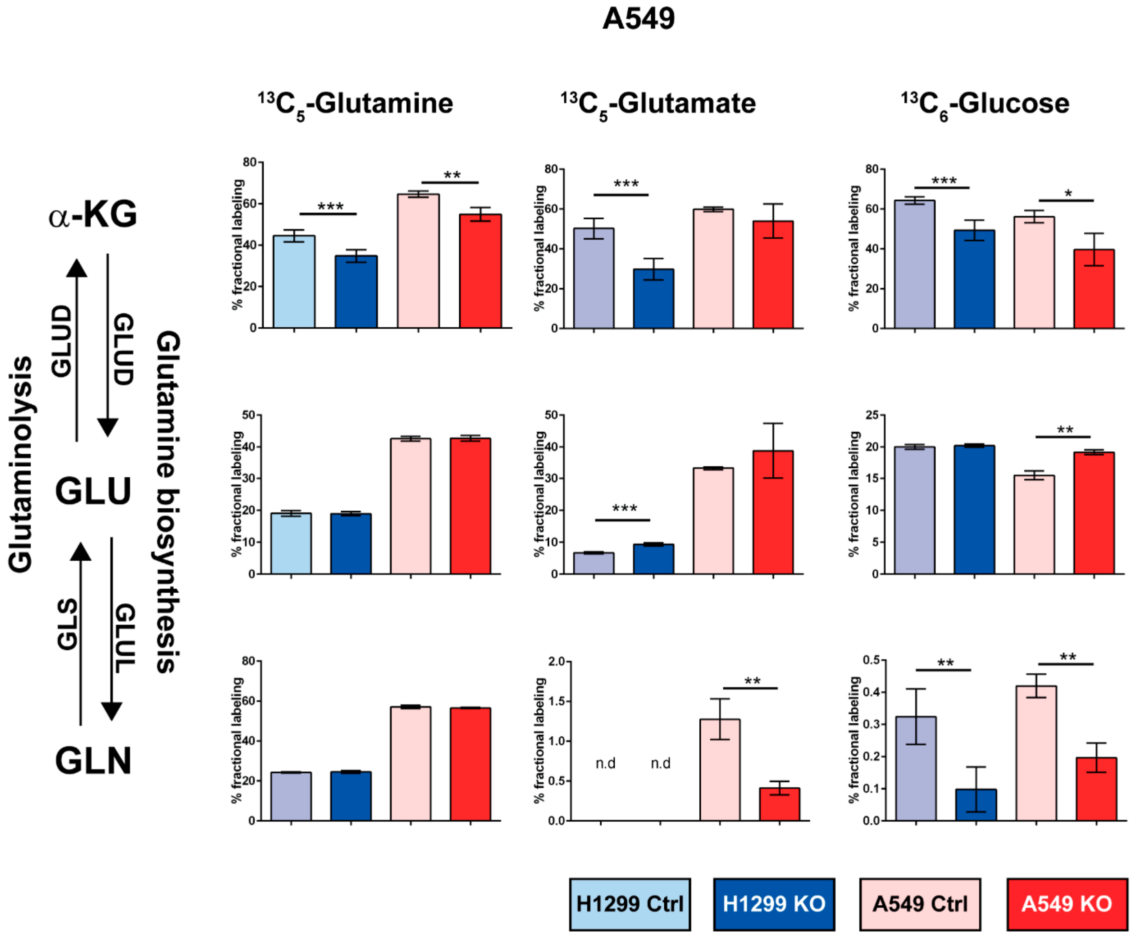

Figure 7. %-Fractional labelling of glutaminolysis intermediates. Glutamine (GLN), Glutamate (GLU) and alpha-ketoglutarate (α-KG). Glutamate dehydrogenase (GLUD), Glutaminase (GLS), Glutamate-ammonia ligase (GLUL).

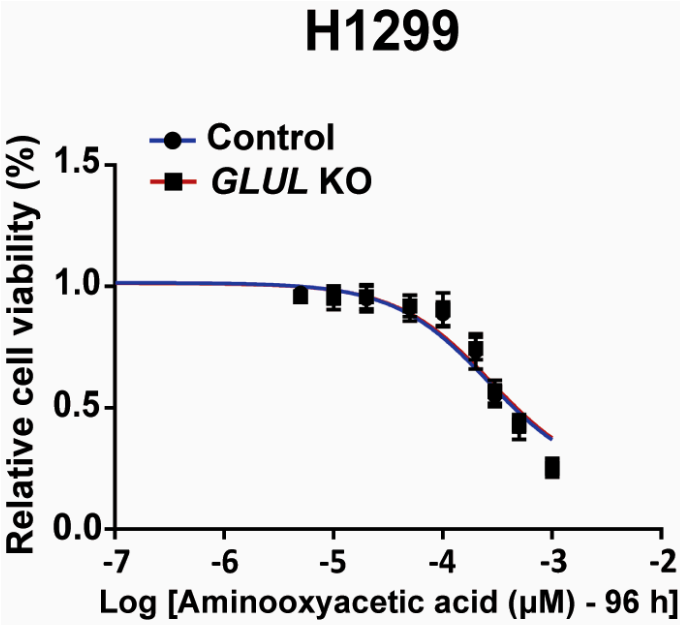

Figure 8. H1299 KO cells do not display sensitivity to ATT inhibition. H1299 control and *GLUL* KO cl3 (Clone 2)cells were either treated with DMSO or treated with inhibitor Aminooxyacetic acid of various concentration and cell viability were analysed by MTS assay after 96 h. Further, IC_{50} were determined as indicated in method section (260 μM). The standard error (SE) bars in cell viability curve represent means of three independent experiments.

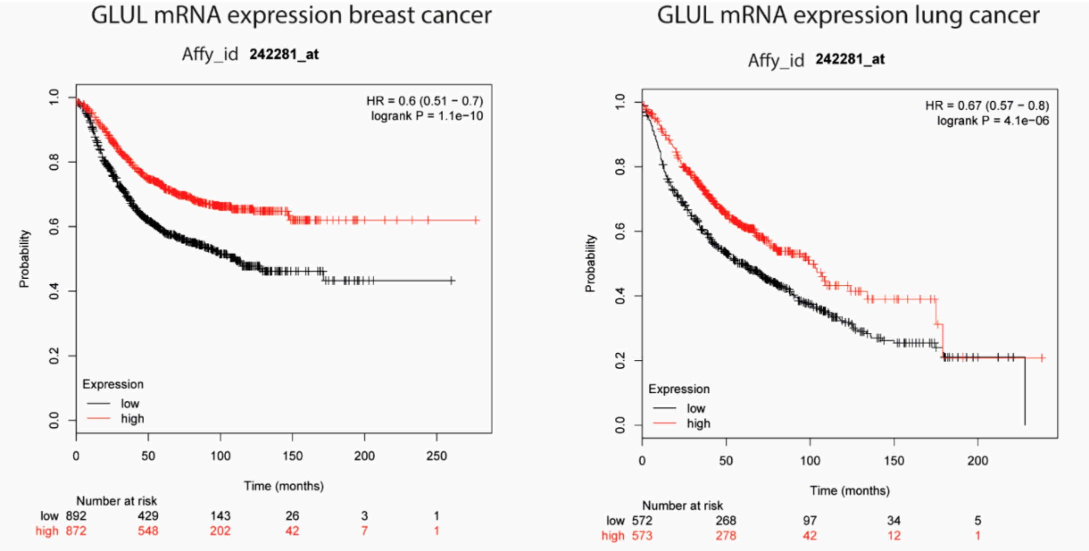

Figure 9. Reduced *GLUL* mRNA expression correlates with poor prognosis in breast and lung cancer patients. The kapalan-Meier Plotter database (http://kmplot.com) was quereid using all default settings in breast cancer and lung cancer patient cohorts containing expression of mRNA and survival data. Black line shows survival for patients with low expression of *GLUL*. Low expression is strongly correlating with poorer survival (p values top right corners of both panels).

Raw blots:

Figure 1A

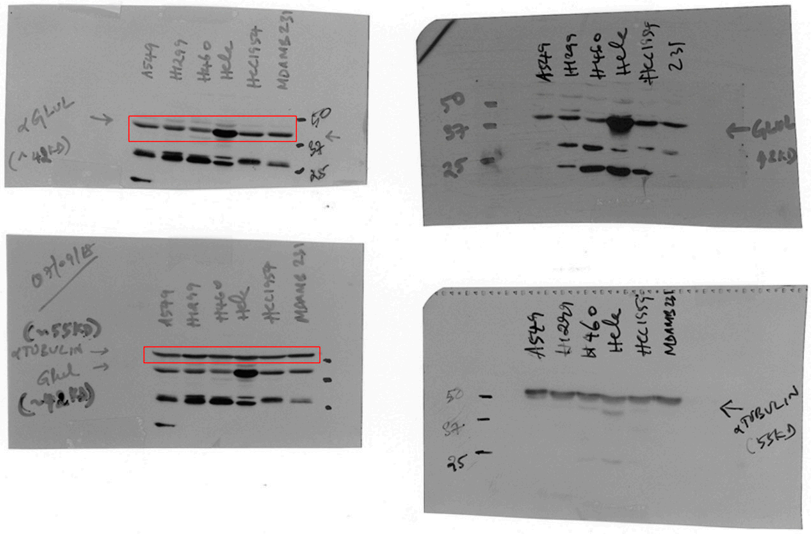

Figure 1B

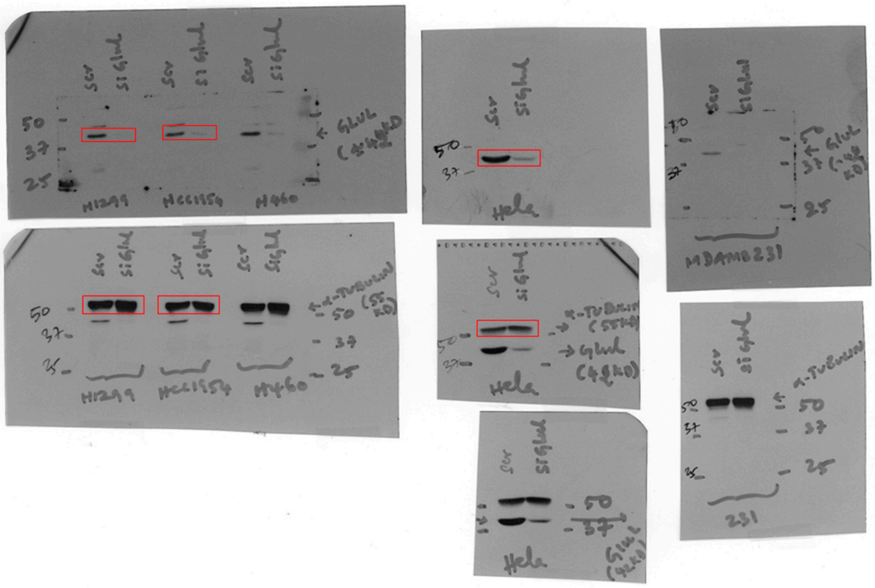

Figure 1B

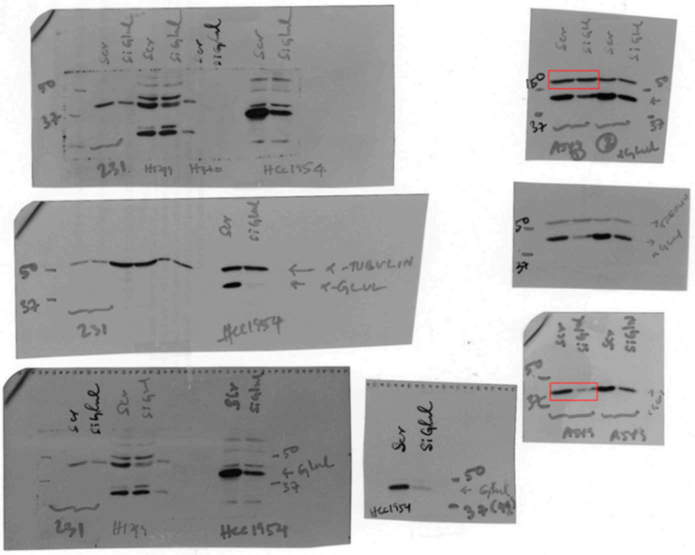

Figure 1B

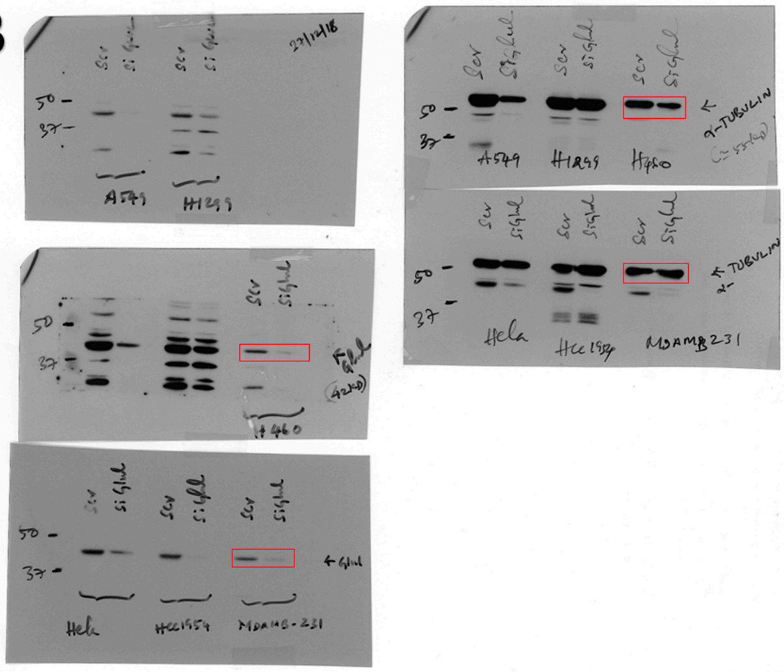

Figure 2A

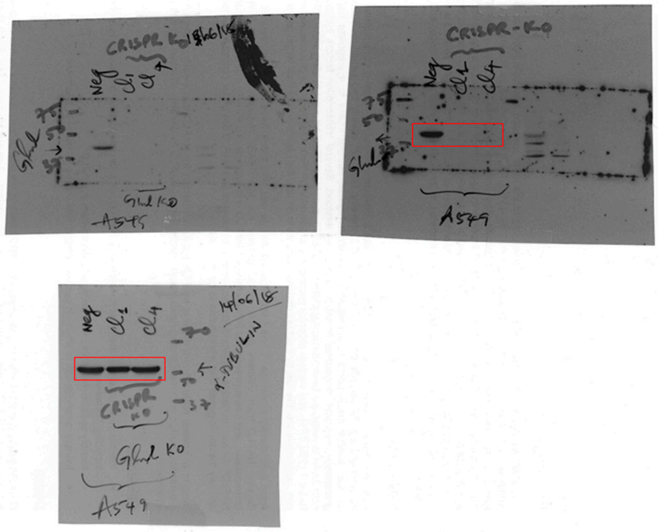

Figure 2A

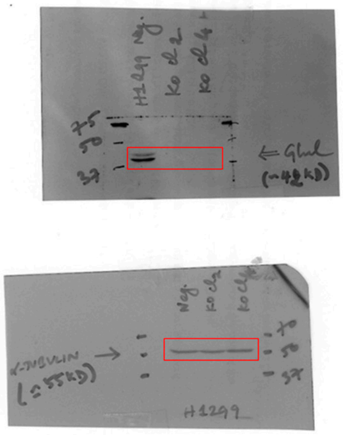

Figure 3

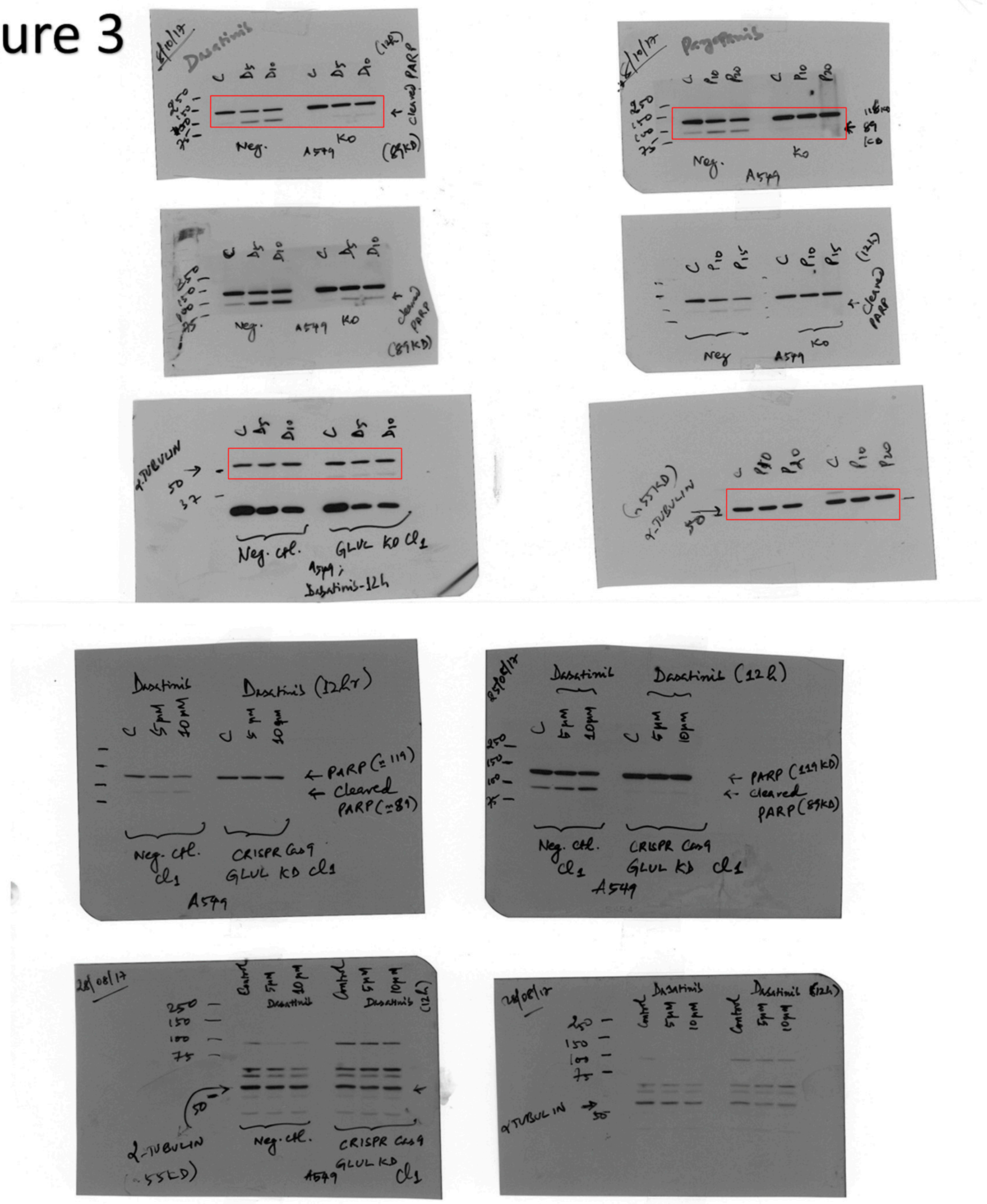
Dasatinib
Pazopanib
Neg.
A549
KO
cleaved PARP
α-TUBULIN
Neg. ctl.
GLUL KD Cl1
Dasatinib (12h)
PARP (119 KD)
cleaved PARP (89KD)
CRISPR Cas9
GLUL KD cl1
Control
5μM
10μM
250
150
100
75
50

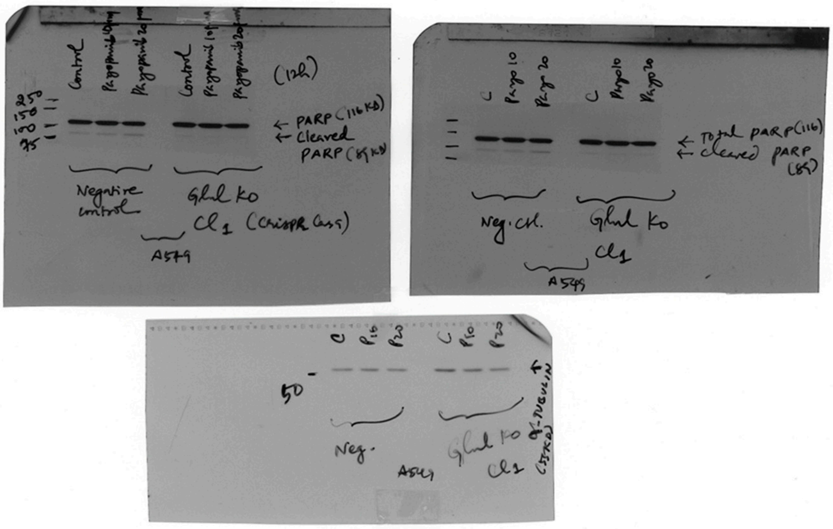

(12h)
Negative control
Glul KO Cl1 (CRISPR Cas)
A549
Neg. Ctl.
Glul KO Cl1
A549

Figure 3

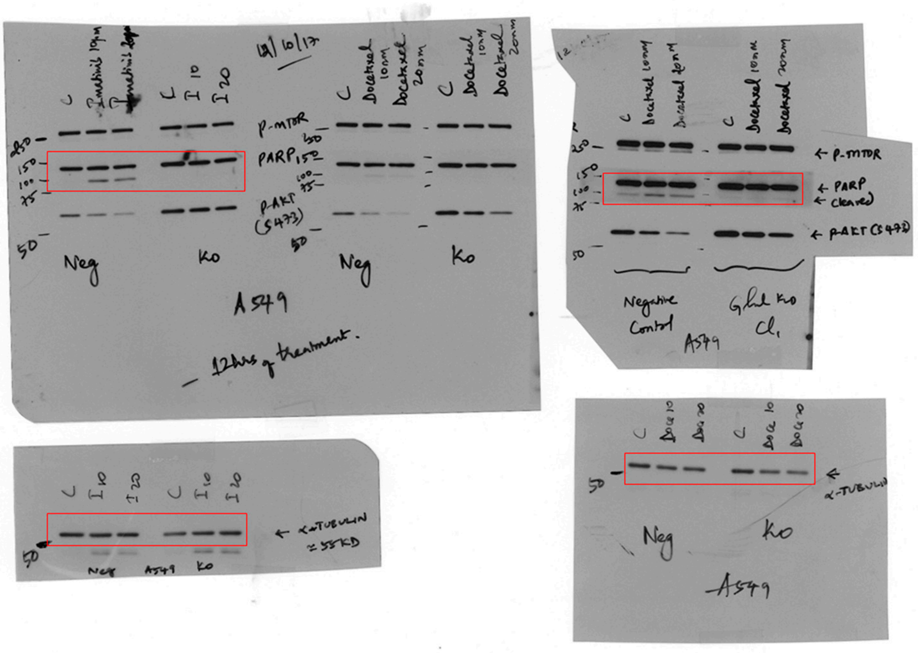

p-mTOR
PARP
p-AKT (S473)
Neg
KO
A549
12 hrs of treatment.
Negative Control
Glul KO Cl1
A549

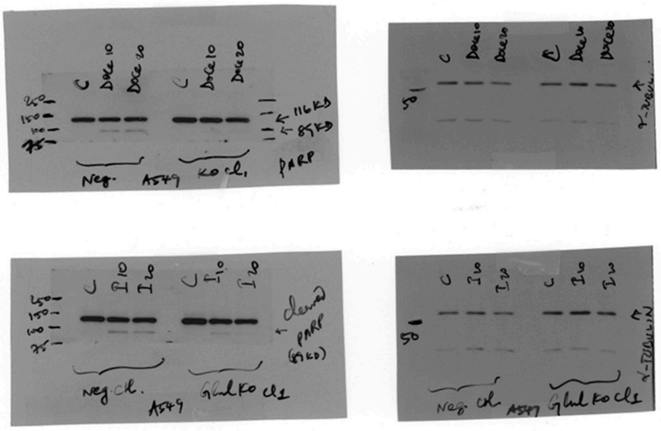

Figure 6C

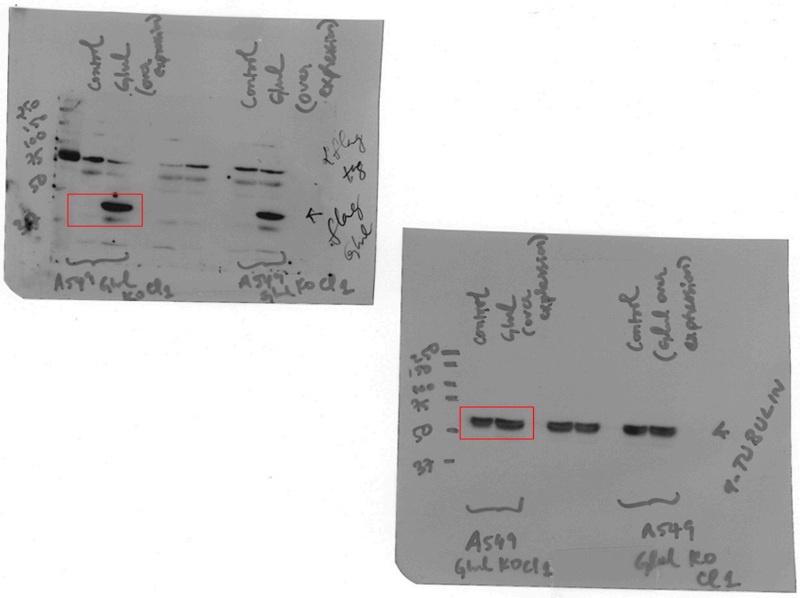

Suppl. Figures:

Figure S1A

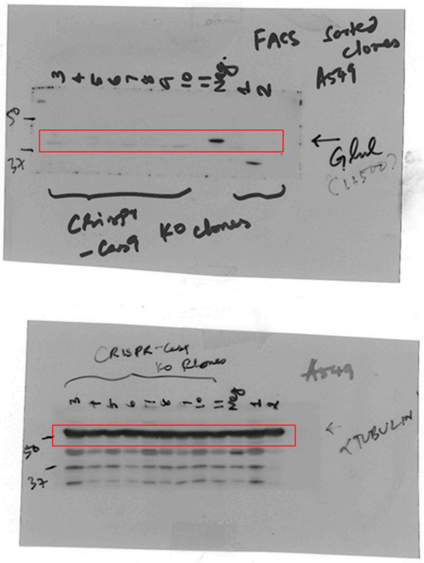

Figure S1B

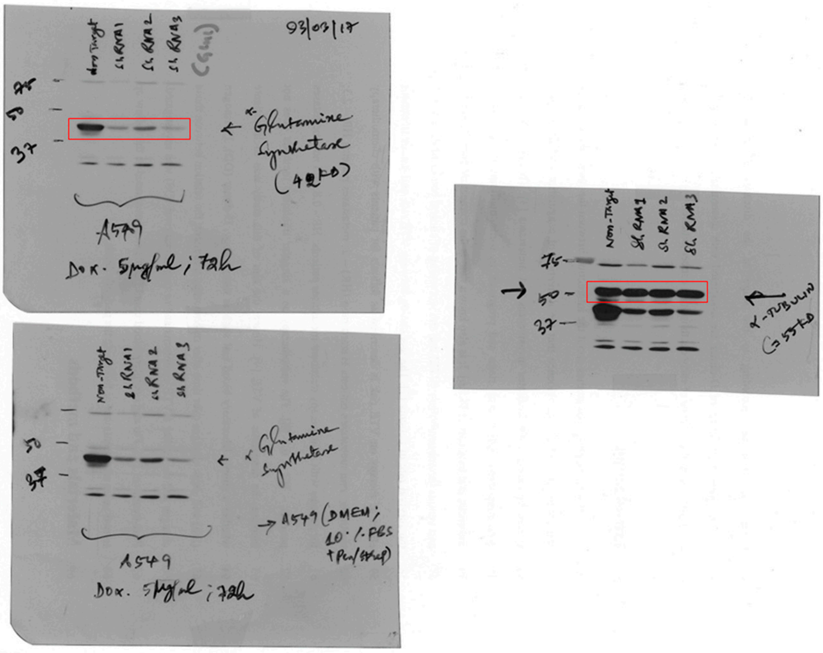

Figure S1C

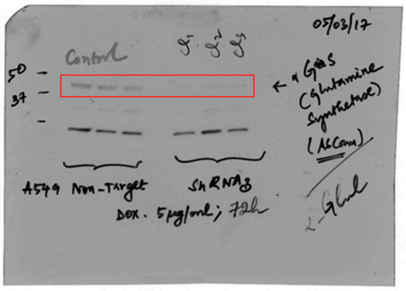

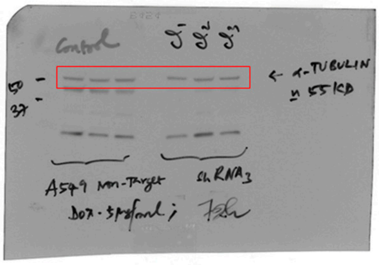

Figure S2C

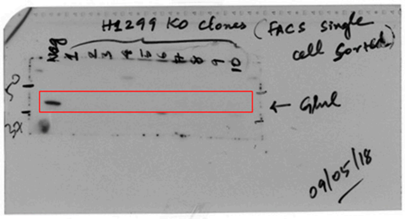

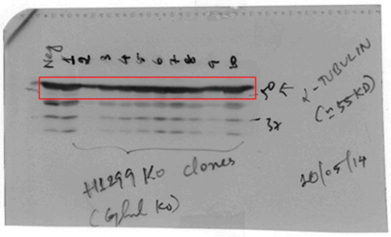

Figure S3

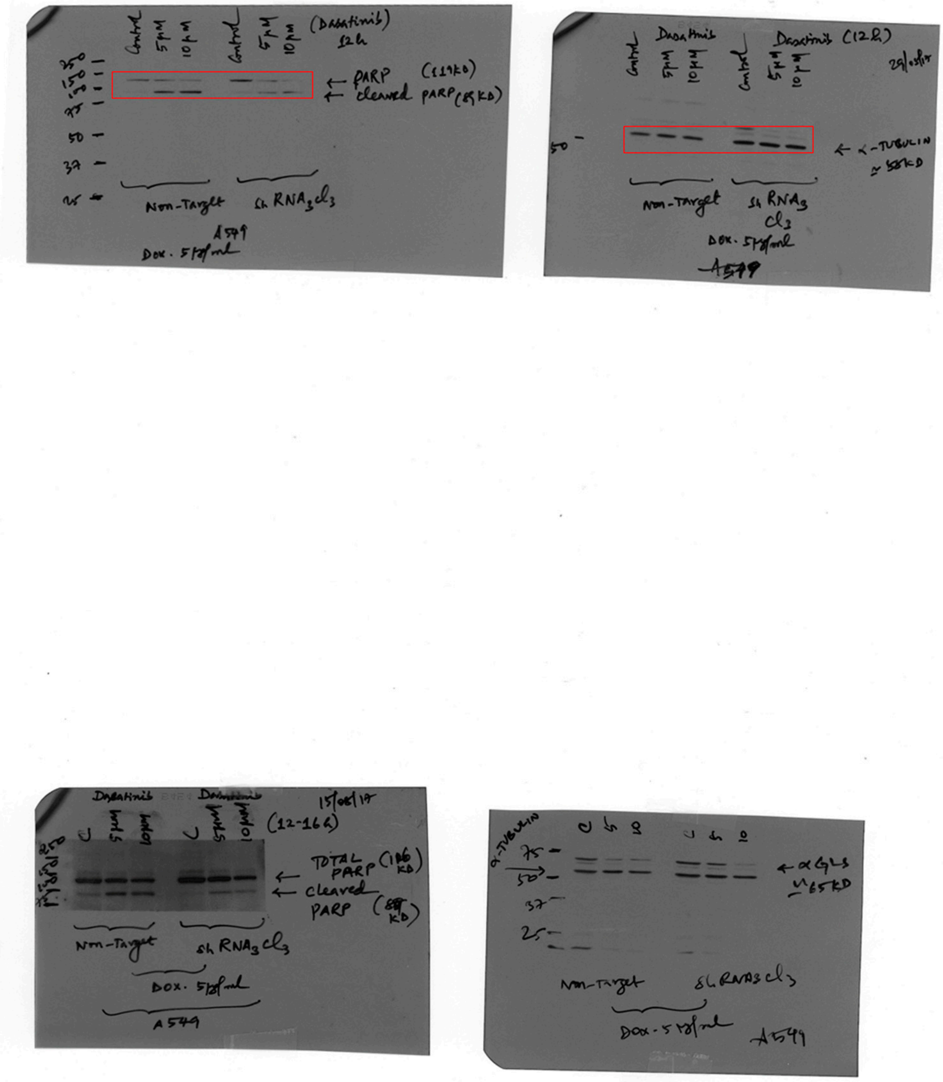

Control
5μM
10μM
(Dasatinib)
12h
PARP
cleaved PARP
Non-Target
sh RNA3 cl3
A549
DOX. 5μg/ml
Dasatinib
Control
α-TUBULIN
Non-Target
sh RNA3
Cl3
DOX. 5μg/ml
A549
TOTAL PARP
cleaved PARP
Non-Target
sh RNA3 cl3
DOX. 5μg/ml
A549
α-TUBULIN
αGLS
Non-Target
shRNA3 cl3
Dox. 5μg/ml
A549

Figure S3

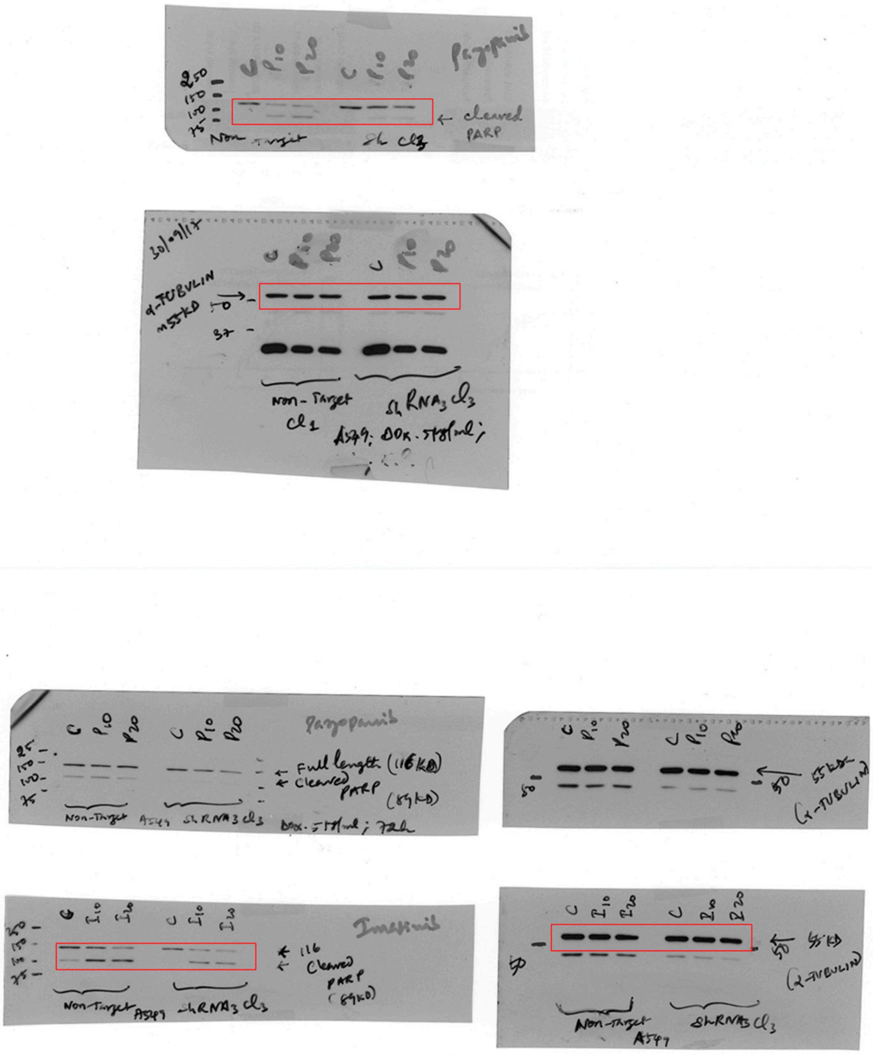
cleaved PARP
Non-Target
sh RNA3 cl3
Full length (116KD)
Cleaved PARP
Non-Target
A549
shRNA3 cl3
Imatinib
Cleaved PARP

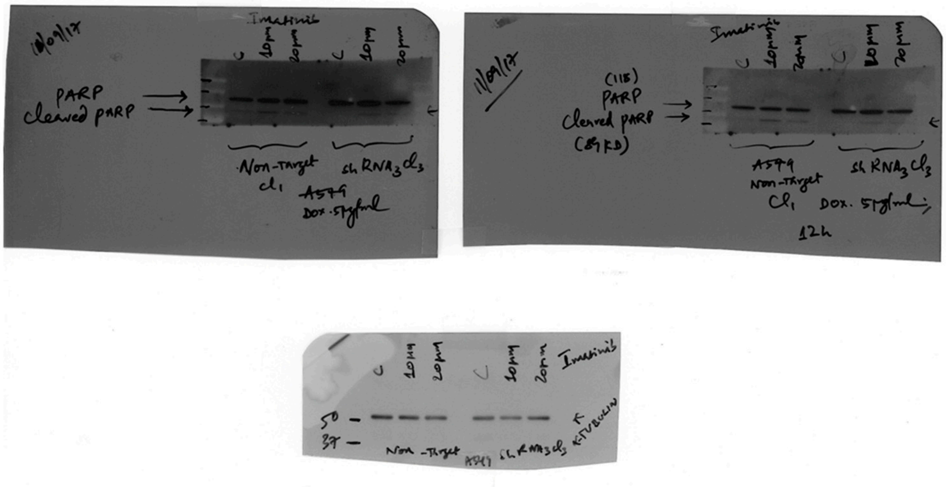
Imatinib
PARP
Cleaved PARP
Non-Target
A549
Dox. 5 μg/ml
12h

Figure S6

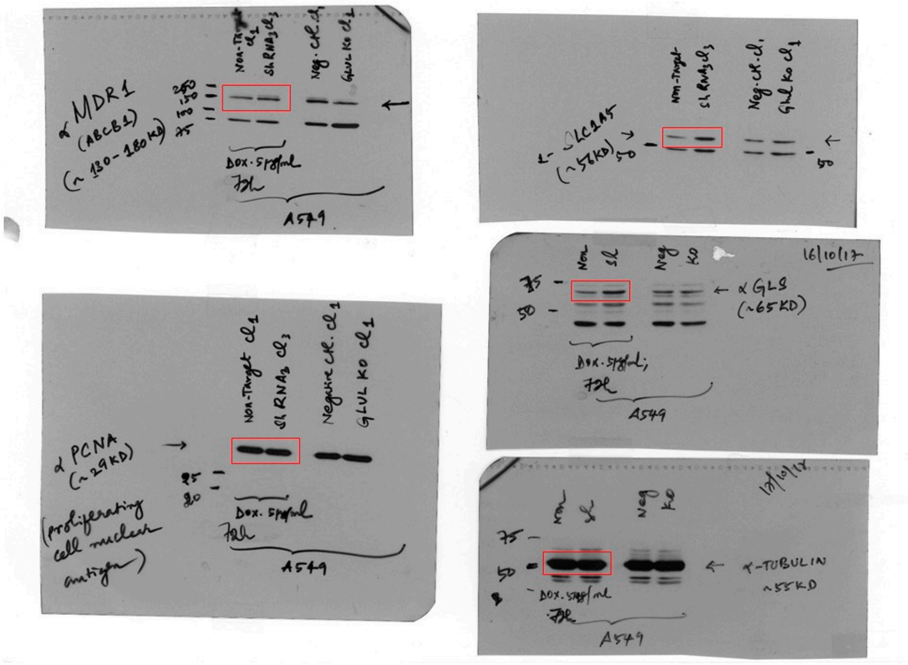
α MDR1
(ABCB1)
(~130-180 KD)
Non-Target
Neg. ctr. cl1
GLUL KO cl1
Dox. 5 μg/ml
72h
A549
α GLS
(~65 KD)
α PCNA
(~29 KD)
(proliferating cell nuclear antigen)
α-TUBULIN
~55 KD
